# Crystal‐storing histiocytes and plasma cells, crystalline corneal deposits and atypical MRI lesions in multiple myeloma

**DOI:** 10.1002/jha2.530

**Published:** 2022-08-04

**Authors:** Muhammad Khakwani, Hayder Hussein, Guy Pratt, Steve Amerasekera, Juliette Edwards, Sai Kolli

**Affiliations:** ^1^ Department of Haematology University Hospital of Birmingham NHS Foundation Trust Birmingham UK; ^2^ Department of Radiology University Hospital of Birmingham NHS Foundation Trust Birmingham UK; ^3^ Department of Ophtalmology University Hospital of Birmingham NHS Foundation Trust Birmingham UK

1

A 46‐year‐old male presented to his General Practitioner (GP) feeling generally unwell with bony aches and blurriness of vision. Routine blood tests performed by the GP showed acute kidney injury with creatinine of 121 μmol/L. Subsequent blood tests performed at the renal clinic showed a mild anemia of 125 g/L and an IgG kappa paraprotein of 17.2 g/L. The rest of his full blood count, renal and calcium profiles were within the normal ranges. A bone marrow examination showed a 35% population of neoplastic plasma cells, which were cytoplasmic kappa light chain restricted and positive for CD38, CD138, CD56, and CD200 confirming the diagnosis of myeloma. The bone marrow morphology was remarkable for crystal‐storing histiocytes and plasma cells (Figure 1A,B at objective ×100). An ophthalmology examination revealed crystalline corneal deposits and anterior chamber inflammation, with complete corneal stroma infiltration bilaterally making corneae appear hazy on slit lamp examination (Figure 2). Whole spine specialized magnetic resonance imaging (MRI) sequences showed unusual low signal osseous deposits (Figure 3) without hypersensitivity, which were atypical for lytic lesions, but suspicious for paraprotein crystalline deposition.

**FIGURE 1:**
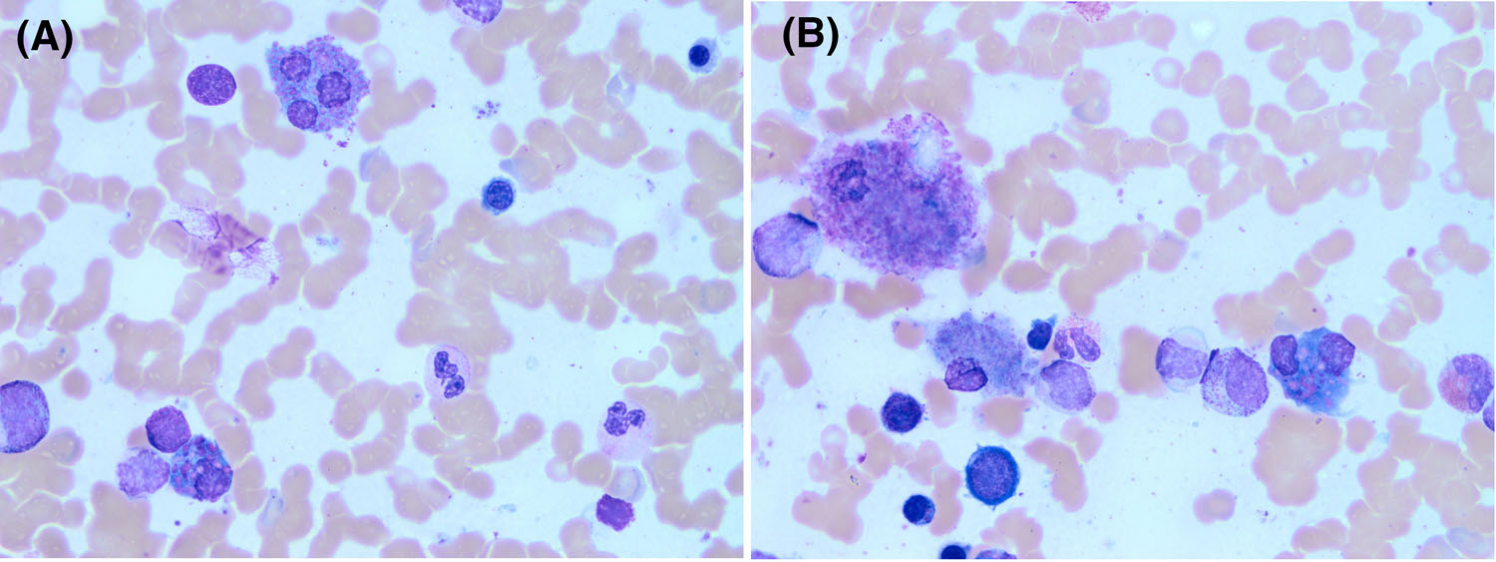
Crystal deposition seen in plasma cells and histiocytes in bone marrow

**FIGURE 2:**
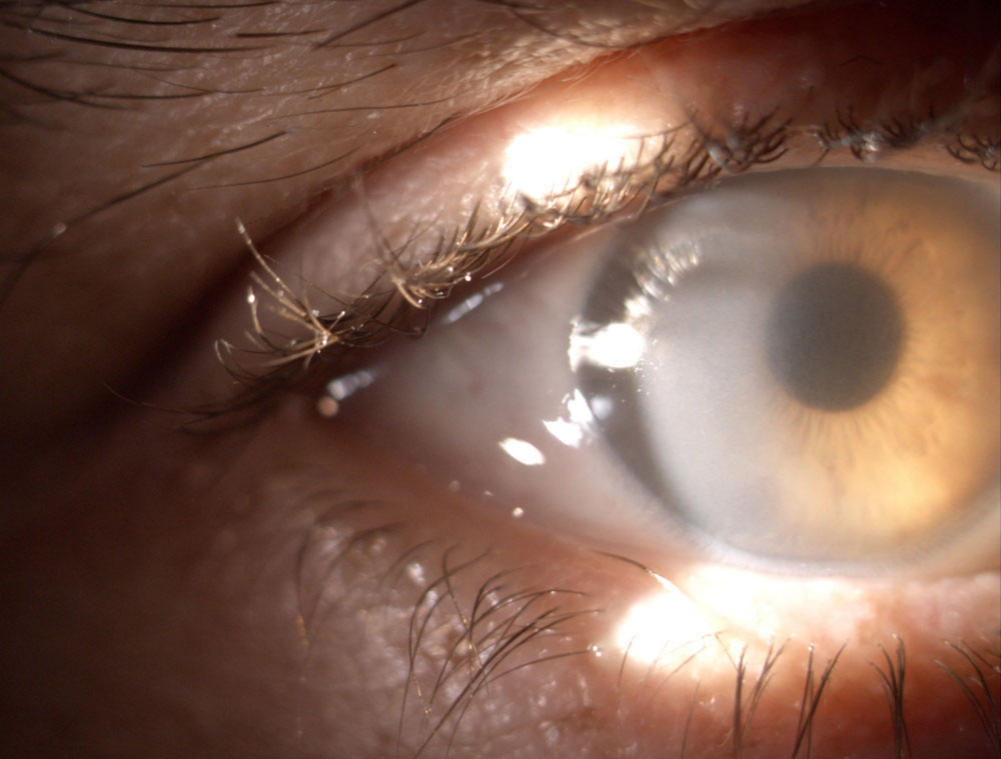
Slit lamp view of right eye showing complete stromal infiltration and hazy cornea

**FIGURE 3:**
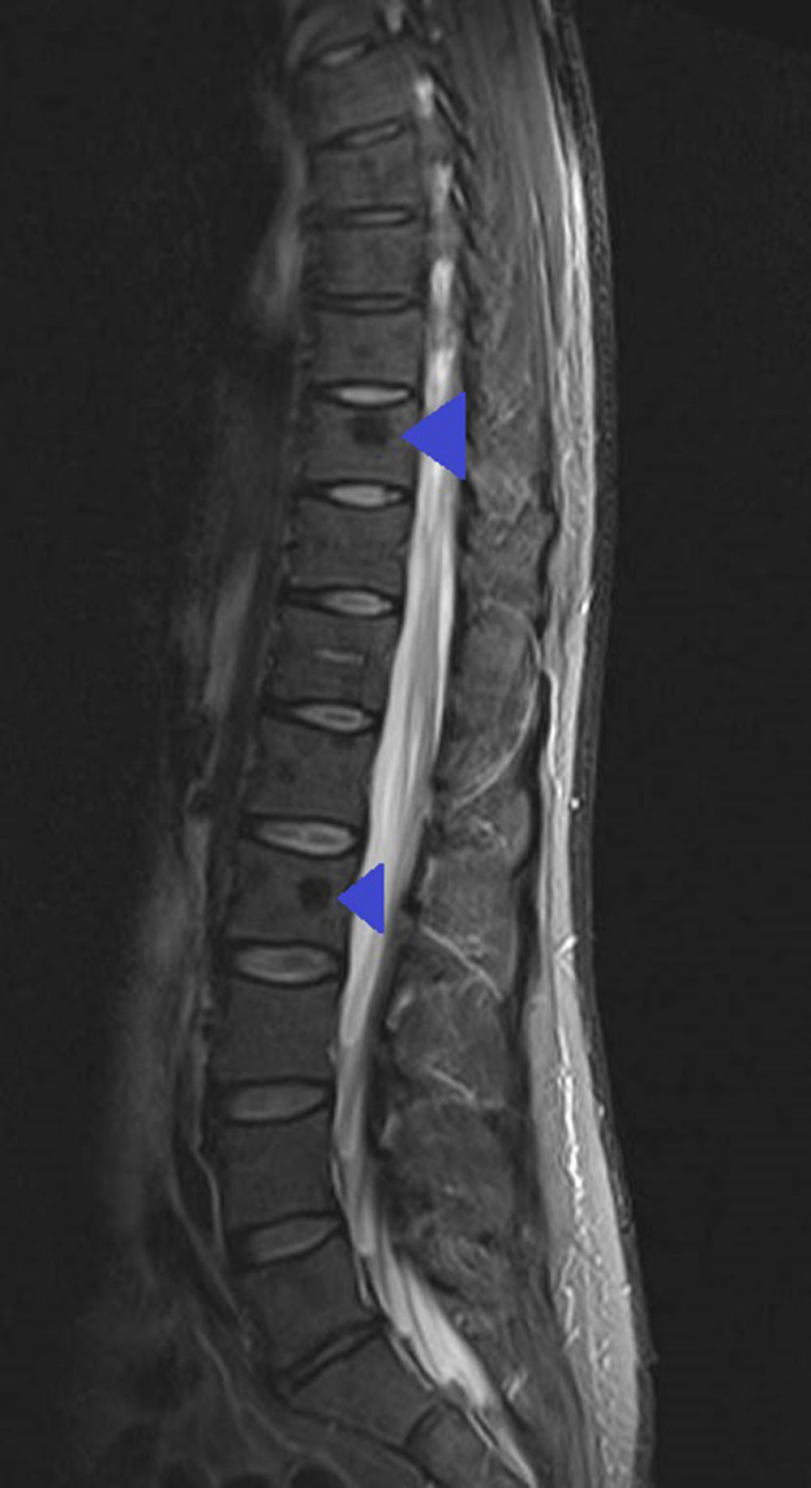
Magnetic resonance imaging (MRI) thoracic and lumbar spine demonstrating low signal lesions in the T10 and L2 vertebral bodies

The patient received bortezomib, thalidomide, dexamethasone and daratumomab combination chemotherapy with topical steroids for the anterior chamber inflammation, with improvement in his paraprotein level and normalization of his renal function, with a plan for autologous stem cell transplant for consolidation.

This case highlights a rare presentation of multiple myeloma with crystal‐storing histiocytes associated with atypical spine MRI changes and crystalline corneal deposits at presentation of disease.

## CONFLICT OF INTEREST

The authors declare no conflict of interest.

## FUNDING INFORMATION

The authors received no funding for this study.

## PATIENT CONSENT STATEMENT

Patient gave his verbal consent for the publication and there is no patient indefinable material used for the article.

## ETHICS STATMENT

The article represents the original work contributed by all the authors from University Hospital of Birmingham.

## Data Availability

The authors are convinced for data sharing as per the recommendations of the journal.

